# Lymphotoxin expression in human and murine renal allografts

**DOI:** 10.1371/journal.pone.0189396

**Published:** 2018-01-04

**Authors:** Harald Seeger, Maja T. Lindenmeyer, Clemens D. Cohen, Carsten Jaeckel, Peter J. Nelson, Jin Chen, Ilka Edenhofer, Nicolas Kozakowski, Heinz Regele, Georg Boehmig, Simone Brandt, Rudolf P. Wuethrich, Mathias Heikenwalder, Thomas Fehr, Stephan Segerer

**Affiliations:** 1 Division of Nephrology, University Hospital, Zuerich, Switzerland; 2 Institute of Physiology and Zuerich Center for Integrative Human Physiology (ZIHP), University of Zurich, Zuerich, Switzerland; 3 Nephrological Center, Medical Clinic and Policlinic IV, University of Munich, Munich, Germany; 4 Clinical Institute of Pathology, University of Vienna, Vienna, Austria; 5 Division of Nephrology and Dialysis, Department of Medicine III, Medical University Vienna, Austria; 6 Institute of Surgical Pathology, University Hospital Zuerich, Zurich, Switzerland; 7 Division of Chronic Inflammation and Cancer, German Cancer Research Center (DKFZ), Heidelberg, Germany; 8 Department of Internal Medicine, Kantonsspital Graubuenden, Chur, Switzerland; 9 Division of Nephrology, Kantonsspital Aarau, Aarau, Switzerland; George Washington University School of Medicine and Health Sciences, UNITED STATES

## Abstract

The kidney is the most frequently transplanted solid organ. Recruitment of inflammatory cells, ranging from diffuse to nodular accumulations with defined microarchitecture, is a hallmark of acute and chronic renal allograft injury. Lymphotoxins (LTs) mediate the communication of lymphocytes and stromal cells and play a pivotal role in chronic inflammation and formation of lymphoid tissue. The aim of this study was to assess the expression of members of the LT system in acute rejection (AR) and chronic renal allograft injury such as transplant glomerulopathy (TG) and interstitial fibrosis/tubular atrophy (IFTA). We investigated differentially regulated components in transcriptomes of human renal allograft biopsies. By microarray analysis, we found the upregulation of LTβ, LIGHT, HVEM and TNF receptors 1 and 2 in AR and IFTA in human renal allograft biopsies. In addition, there was clear evidence for the activation of the NFκB pathway, most likely a consequence of LTβ receptor stimulation. In human renal allograft biopsies with transplant glomerulopathy (TG) two distinct transcriptional patterns of LT activation were revealed. By quantitative RT-PCR robust upregulation of LTα, LTβ and LIGHT was shown in biopsies with borderline lesions and AR. Immunohistochemistry revealed expression of LTβ in tubular epithelial cells and inflammatory infiltrates in transplant biopsies with AR and IFTA. Finally, activation of LT signaling was reproduced in a murine model of renal transplantation with AR. In summary, our results indicate a potential role of the LT system in acute renal allograft rejection and chronic transplant injury. Activation of the LT system in allograft rejection in rodents indicates a species independent mechanism. The functional role of the LT system in acute renal allograft rejection and chronic injury remains to be determined.

## Introduction

Incidence and severity of acute allograft rejection (AR) has been improved with better one-year graft survival, but long-term allograft outcome has lacked behind expectations [1–5]. A better understanding of the inflammatory cascade leading to progressive loss of allograft function will help to define new targets for therapeutic interventions. Cytokines play a significant role in acute and chronic allograft rejection. Chemokines serve as attractants for immune cells and lead to their influx from blood into the allograft. Locally, cytokines stimulate immune cell proliferation, (lymph-) angiogenesis and tissue fibrosis. Conversely, they can exert immune regulatory functions and limit graft inflammation [4, 6–9]. Whereas AR can be treated successfully in the majority of cases, chronic allograft injury such as transplant glomerulopathy (TG) and interstitial fibrosis/tubular atrophy (IFTA) poses a vast problem. Mechanisms leading to chronic allograft injury are diverse. Immunological insults such as repeated acute or chronic cellular or antibody mediated rejection, but also nonimmunological mechanisms such as calcineurin inhibitor toxicity, infections (BK nephropathy, pyelonephritis) or postrenal obstruction might be causative [10]. Even though not considered by the current Banff guidelines, chronically injured grafts with inflammation in the fibrotic and atrophic areas (i-IFTA) have a significantly worse outcome compared to grafts with IFTA alone [11, 12] implicating a deleterious effect of chronic interstitial inflammation. Lymphotoxins (LTs) are important players in acute and chronic inflammation, yet their contribution to kidney allograft injury has not been thoroughly investigated. LTs are members of the TNF family consisting of the ligands lymphotoxin α (LTα), β (LTβ), tumor necrosis factor (TNF), and LIGHT (TNSF14). The homotrimer Ltα3 signals via TNF receptors (TNFRs) 1 (and 2) whereas the heterotrimer LTα1β2 only binds to the lymphotoxin β receptor (LTβR). LIGHT has specificity for the receptors HVEM (Herpes virus entry mediator), LTβR and DcR3 (reviewed in [13]). LTβR is strongly expressed in the mouse and human kidney [14, 15] but also by other parenchymal and endothelial cells [16–19]. Activation via LIGHT or LTα1β2 triggers NF-kB pathways [20]. LT signaling plays a central role in chronic inflammation and the formation of tertiary lymphoid organs (TLOs) [21–23]. In renal allograft biopsies TLOs are present in up to 50% [24]. Even though early studies have provided evidence for the expression of LTβ in rejected rat and human renal allografts [25, 26], a comprehensive investigation regarding the expression of LTs in kidney allografts has not been undertaken. The aim of this study was to examine the differential regulation of components of the LT system in human renal allograft biopsies and a murine model of renal transplantation.

## Material and methods

### In silico analysis of LT expression

In a first analysis, microarray data from the public domain were used (GEO database: http://www.ncbi.nlm.nih.gov/geo; project GSE9493; Affymetrix HG-U133Plus2.0 arrays). This project includes mRNA expression data from human renal biopsies with AR (n = 8), chronic allograft injury (interstitial fibrosis and tubular atrophy, IFTA, n = 21) and controls (n = 12 from renal cancer nephrectomy samples, nonaffected renal cortex) [27, 28]. We have summarized clinical characteristics of these patients in Table 1 and S1 Table. CEL file normalization was performed with the Robust Multichip Average method and the human Entrez-Gene custom CDF annotation from Brain Array version 15 (http://brainarray.mbni.med.umich.edu/Brainarray/default.asp) using RMAExpress (Version 1.0.5). We defined a background filter cut-off using the highest signal value obtained from a nonhuman Affymetrix-control oligonucleotide multiplied by a factor of 1.2, corresponding in the current dataset to a log-based 2 value of 7.35. Subsequently we analyzed the differential expression with Significance Analysis of Microarrays (SAM) using TiGR (MeV, Version 4.8.1; http://www.tm4.org/mev/) [29]. The main results were also confirmed in a single probe analysis approach (ChipInspector, Genomatix, Munich, Germany).

**Table 1:** Basic clinical parameters of the patients included from the public domain (GEO database) for analysis of lymphotoxin expression in the transcriptome of graft biopsies. (n = number, m = male, f = female, nd = not determined).

Diagnosis	n	Gender (m/f)	Mean age in years (range)	Creatinine in μmol/l (range)
Acute rejection	8	6/2	44 (22–54)	253 (161–466)
IFTA	21	15/6	48.6 (24–67)	287 (110–1000)
Controls	12	9/2/nd	55.8 (17–82)	85 (36–114)

### Lymphotoxin and related gene expression in renal allografts with transplant glomerulopathy

For this analysis mRNA from human renal biopsy specimens, collected in an international multicenter study, the European Renal cDNA Bank-Kröner-Fresenius biopsy bank (ERCB-KFB, see appendix for participating centers) was used [30]. Biopsies were collected after informed consent was obtained and with approval of local ethics committees. None of the transplant donors was from a vulnerable population and all donors or next of kin provided written informed consent that was freely given. Samples and clinical data were analyzed anonymously. Kidney biopsies of patients with transplant glomerulopathy (TG; n = 14) from the ERCB-KFB and controls (n = 4; donor kidneys prior to implantation) were microdissected. mRNA was extracted from the glomerular and the tubular compartment as per standard protocols, hybridized to HG-U 133 Plus 2.0 cDNA microarrays (Affymetrix) and the intensities measured as per standard protocols. We carried out quality control and clustering analyses using the MADMAX (Management and Analysis Database for Multi-platform microArray eXperiments) platform (https://madmax.bioinformatics.nl, University of Wageningen). This lead to a distinct separation of the TG samples into two clusters. The same platform was used to normalize the data using the RMA algorithm [31]. Normalized intensities for each gene were then expressed as a fraction of the maximum intensity for each gene (i.e. setting the highest expression value to 1). Heatmaps were drawn using Multi experiment Viewer 4.8.1 (http://www.tm4.org/mev/) applying the Pearson clustering strategy. Table 2 and S2 Table display the basic clinical parameters of the patients with transplant glomerulopathy included in the microarray analysis.

**Table 2:** Basic clinical parameters of the patients with transplant glomerulopathy included in the microarray analysis. (n = number, m = male, f = female, nd = not determined, TG = transplant glomerulopathy).

Diagnosis	n	Mean age in years (range)	Recipient sex	Creatinine in μmol/l (range)	Proteinuria in g/day (range)
**TG**	n	48 (37–68)	m/f/nd 6/4/4	256 (143–512)	3.3 (0,0–7.6)
**Controls**	14	48 (25–61)	m/f 0/4	nd	nd

### Quantitative real-time RT-PCR (qPCR)

For mRNA expression analysis by RT-PCR renal biopsy specimens from ERCB-KFB [30] were used (see above). Tissue was transferred to RNase inhibitor immediately after the biopsy was taken. Total RNA was isolated according to a protocol previously reported [30]. Pre-developed TaqMan reagents were used for human LTα (Hs00236874_m1), LTβ (Hs00242739_m1), LTβ-receptor (Hs00158922_m1), LIGHT (Hs00542477_m1), DcR3 (Hs00187070_m1) and HVEM (Hs00998604_m1) and the housekeeper genes GAPDH and 18SrRNA (Applied Biosystems, Darmstadt, Germany). mRNA expression was analyzed by standard curve quantification. Included in the study were biopsies with borderline lesions (n = 10), AR (n = 22), and signs of chronic injury (IFTA, n = 7). Control biopsies were taken from living donors before implantation (n = 10). Clinical data of these patients is summarized in Table 3 and S3 Table.

**Table 3:** Basic clinical parameters of the patients included from the ERCB-KFB cohort for RTPCR expression analysis. (n = number, m = male, f = female, nd = not determined, AR = acute rejection, IFTA = interstitial fibrosis and tubular atrophy, Pre-Tx = pretransplant).

Diagnosis	n	Sex (m/f)	Mean age in years (range)	Time after transplantation in months (range)	Creatinine in μmol/l (range)
Borderline	10	7/3	40 (25–66)	3.8 (0–24)	332.8 (136–919)
AR	22	16/6	52 (38–71)	5.3 (0–48)	217 (177–909)
IFTA	7	4/1/3 nd	52 (42–71)	73.5 (12–180)	294 (120–548)
Pre-Tx	10	5/4/1 nd	48 (27–70)	-	<97

For the correlation between histopathological changes and the mRNA expression levels of cytokines and receptors two observers (S.B. and H.S.) blinded to the histological diagnosis of the initial evaluation reviewed the original biopsy reports of the 39 patients. The histology slides were not available for direct review. We extracted the following histopathological characteristics: atii = acute tubulointerstitial inflammation (interstitial mononuclear infiltration and tubulitis), ctii chronic tubulointerstitial inflammation (interstitial fibrosis and tubular atrophy), ag = acute glomerulitis, cg = chronic glomerulitis (transplant glomerulopathy), av = acute intimal arteritis, cv = chronic intimal arteritis (arterial fibrous intimal thickening), mm = mesangial matrix increase. We also tried to extract information about peritubular capillaritis, however this parameter was not mentioned in most of the reports. Thus, we omitted it from the analysis. In addition, we could not extract sufficient information about C4d peritubular capillary staining, arteriolar hyalinosis and peritubular capillary basement membrane multilayering. We scored a 0 if the report said “none”, “discrete”, “minimal” or “isolated”. We scored a 1 if the report said “low” or “moderate” and a 2 if the report said “strong” or “massive”. The interobserver correlation was excellent (Spearman’s r between 0.954–1). For correlation between the mRNA expression level and a histopathological change, we used Spearman rank correlation analysis.

### Immunohistochemical localization of LTβ in human renal allograft biopsies

We performed immunohistochemistry as previously described [15, 32]. In brief, sections were dewaxed in xylene, rehydrated in a graded series of ethanol, and incubated in 3% hydrogen peroxide. Avidin (Vector, Burlingame, CA) was used to block endogenous biotin. Autoclaving was used for heat based antigen retrieval in antigen retrieval solution (Vector). The primary reagent used was a mouse monoclonal antibody against human LTβ (Clone: B-27 gift from Biogen, Cambridge, MA, USA) in a 1: 500 dilution. Incubation with primary antibody was performed overnight. Incubation with biotinylated secondary antibody (Vector) for 30 minutes was followed by ABC reagent (Vector). 3’3’Diaminobenzidine (DAB, Sigma, Taufkirchen, Germany) with metal enhancement was used as a detection system. A weak methyl green counterstain of the nuclei was used.

Staining was performed on biopsies from 39 kidney transplants with a histologic diagnosis of acute cellular rejection (n = 14), acute humoral rejection (n = 6), mixed acute cellular and humoral rejection (n = 5) and (n = 4) with IFTA. Ten biopsies were implant biopsies which all showed normal histology except for one biopsy with mild interstitial fibrosis/tubular atrophy. Semiquantitative evaluation was performed in a blinded fashion. Slides were scored 0 = (no staining), 1 (positive cells present), 2 (numerous positive cells). Since LTβ expression was only observed in tubular epithelial cells, diffusely infiltrating inflammatory cells and inflammatory cells in follicular infiltrates, these three parameters were quantified. Table 4 and S4 Table display the basic clinical parameters of the patients, which were included in the histological analysis. Retrospective analysis of the transplant biopsies was approved by the ethics committee of the University of Vienna, Austria (permit number 1391/2012).

**Table 4:** Basic clinical parameters of patients from which allograft biopsies were stained for LTβ. (n = number, m = male, f = female, nd = not determined, AR = acute rejection, IFTA = interstitial fibrosis and tubular atrophy).

Diagnosis	n	Mean age in years (range)	Recipient sex	Time after transplantation in months (range)
**Control**	10	38 (4–65)	m/f: 7/3	0,0 (0.0–0.0)
**IFTA**	4	45 (20–77)	m/f: 2/2	46.9 (6.2–126.3)
**AR**	25	49 (20–69)	m/f/nd: 14/10/1	16.0 (0.2–83.2)

### Mouse experiments

#### Renal allografts

All animal experiments were performed according to protocols approved by the legal authorities (Veterinary Office of the Canton of Zuerich; permit number 4888). Mice were housed in specific pathogen-free conditions at the University of Zurich. Kidneys from C57/Bl6 mice were transplanted either into CBA mice (fully MHC mismatched allograft) or into C57/Bl6 mice (isograft controls). Mouse renal allografting was performed as previously described in detail [33]. Surgery was conducted under isoflurane inhalation anesthesia, and all efforts were made to minimize suffering of animals. Mice were euthanized by CO2 inhalation.

#### Routine renal evaluation

Kidneys were fixed in 10% buffered formalin overnight and embedded in paraffin following routine protocols. Paraffin blocks were cut at 3 μm and stained with Haematoxylin-Eosin (H.E.), Periodic Acid Schiff (PAS), and modified Yajima’s Methenamine Silver stain [34].

#### Quantitative RT-PCR

Mouse kidney tissue was immediately transferred to RNAse inhibitor (RNA later) after euthanization and RNA extracted using standard protocols. qRT-PCR was performed as described above using pre-developed TaqMan-assays for mLtα (Mm00440228_gH), mLtβ (Mm00434774_g1), mLtβR (Mm00440228_gH), mTNFRSF14 (HVEM, Mm00619239_m1), mTNFSF14 (LIGHT, Mm00444567_m1) and the housekeeper genes murine β-actin and 18SrRNA (Applied Biosystems, Darmstadt, Germany). mRNA expression was analyzed by the delta delta Ct method.

### Statistics

Data are given as mean ± SEM. Statistical analysis was performed using Kruskal-Wallis and Mann-Whitney U tests (IBM SPSS Statistics 20.0, IBM, Armonk, NY and GraphPad Prism version 5.0 for Windows, GraphPad Software, La Jolla California USA). For correlation analysis, we used Spearman rank correlation. We considered P-values less than 0.05 to indicate statistically significant differences.

## Results

### cDNA microarray analysis of lymphotoxin expression in human renal allograft biopsies with acute and chronic injury

**Fig 1:**
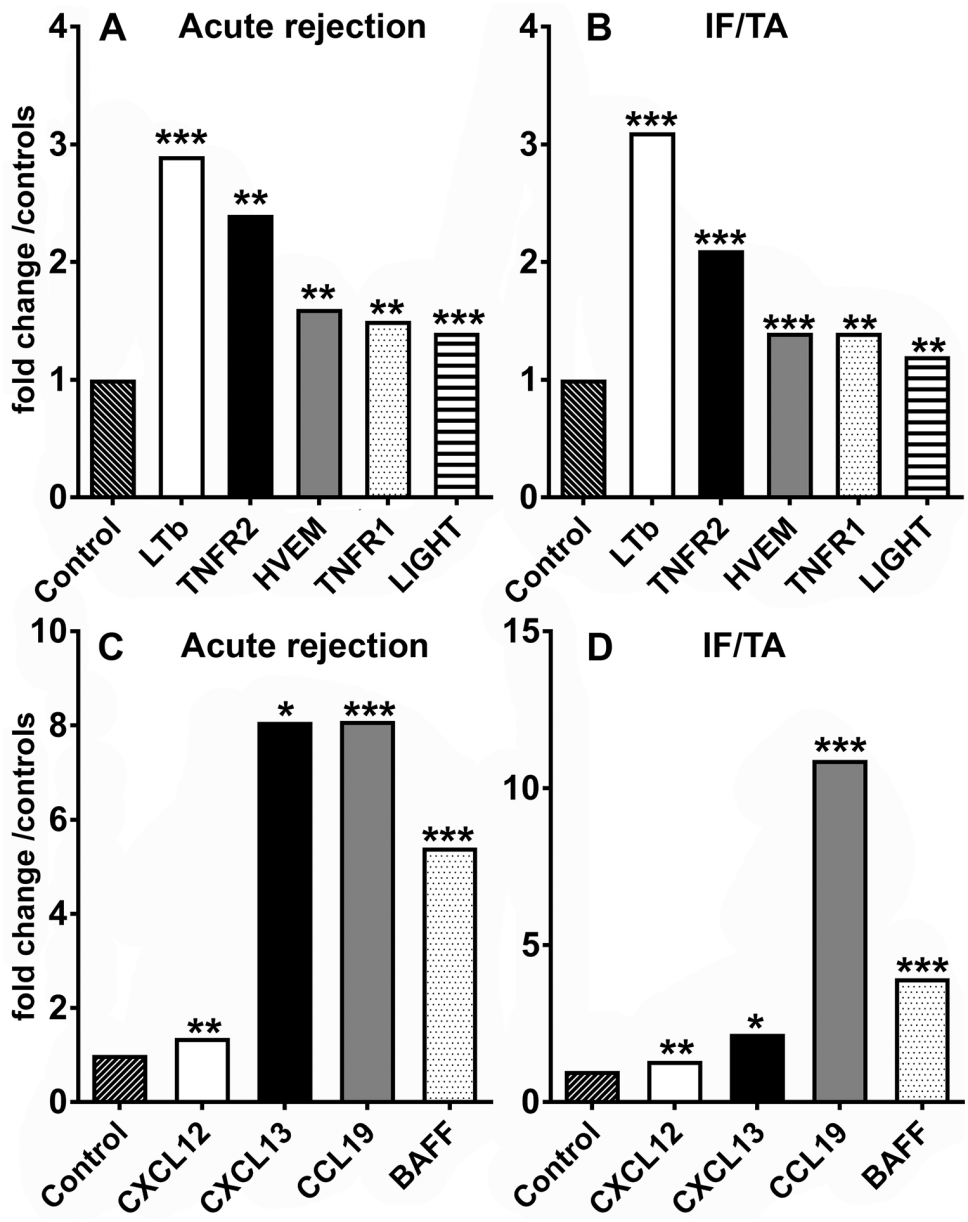
Upregulation of LT and non-canonical NF-κB pathway target transcripts in human renal allograft biopsies with AR and IFTA. LTβ, TNFRs1 and 2, LIGHT and HVEM mRNA expression in human renal biopsies with AR (n = 8; A) or chronic allograft nephropathy (n = 21; B) was significantly increased when compared to controls, which were obtained from nephrectomy specimens after explantation (n = 12). Also, CXCL12, CXCL13, CCL 19 and BAFF showed significant upregulation in AR (C) and IFTA (D). Displayed are fold changes compared to controls (nonaffected renal cortex from renal cancer nephrectomy samples). All fold changes shown were statistically significant compared to controls. (* q-value < 0.05, ** q < 0.01, *** q < 0.001).

To evaluate LT expression in human kidney allografts, we analyzed transcriptome data from 41 human renal allograft biopsies with AR (AR, n = 8), chronic allograft injury (interstitial fibrosis and tubular atrophy, IFTA, n = 21) and nephrectomy controls without rejection (n = 12). Study subjects were predominantly male and the average age at time of biopsy was 44 years in patients with AR, 48 in patients with IFTA and 56 years in control subjects (Table 1 and S1–S3 Tables). In AR and IFTA, LTβ mRNA was significantly upregulated, as was LIGHT and its receptor HVEM (Fig 1A and 1B). The ligands LTα and TNF, as well as LTβR mRNA were expressed, but did not demonstrate a significant induction or downregulation (not illustrated). Yet, expression of TNF receptor 1 and 2 mRNAs (the receptor for LTα3) were increased significantly (Fig 1A and 1B). In addition to transcripts regulated by the canonical NF-kB pathway known targets of the alternate LTβR signaling pathway, such as CXCL12 (SDF-1α), CXCL13 (BLC), CCL19 (ELC) and BAFF were strongly upregulated (Fig 1C and 1D).

### Lymphotoxin mRNA expression patterns in human renal allografts with transplant glomerulopathy

**Fig 2:**
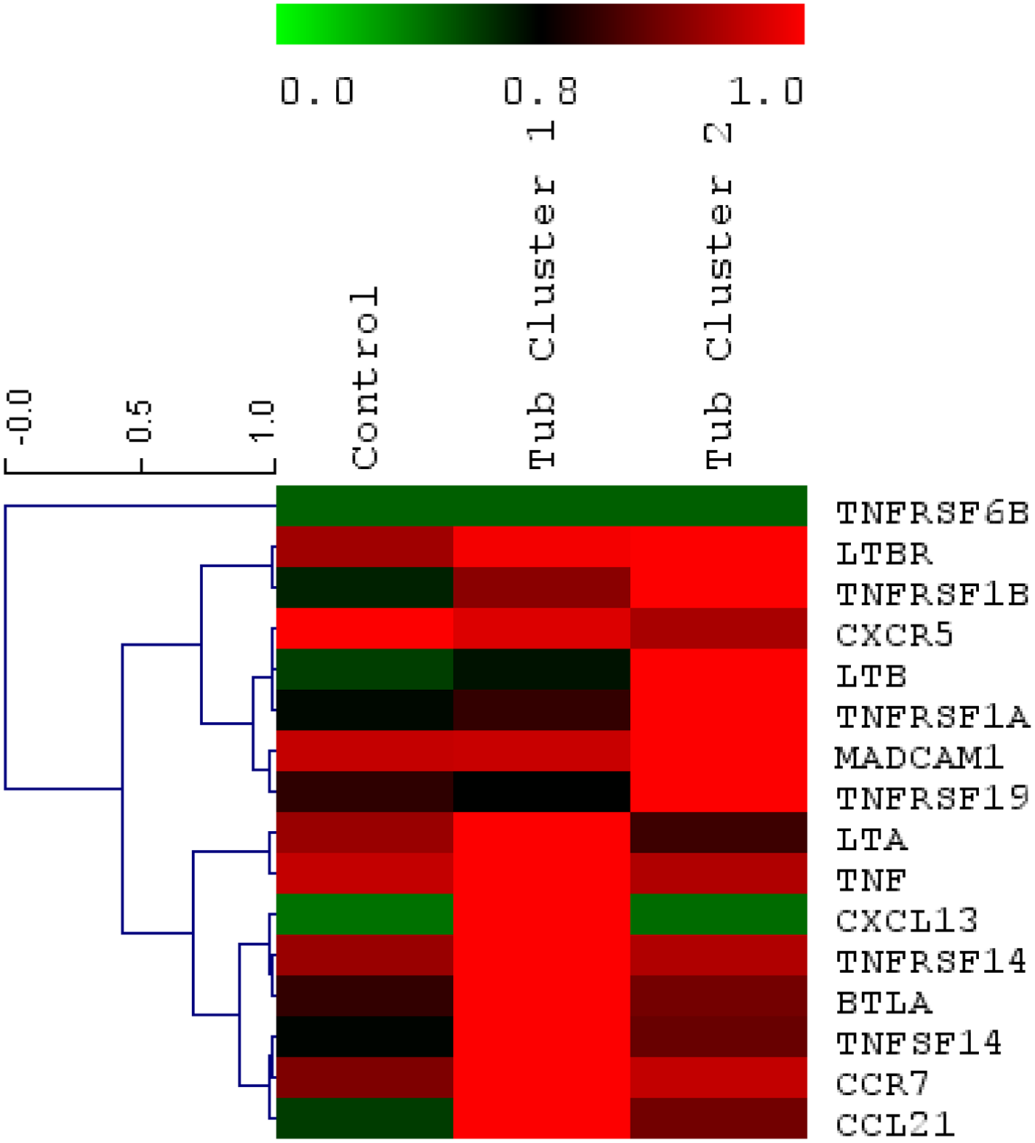
Clustered heatmap of the gene set influenced by lymphotoxin from array data. Samples (x-axis) show transplant glomerulopathy clusters 1 and 2 in comparison to control kidneys. Genes (y-axis) are clustered and colored based on their relative expression levels. The color scale is a double gradient, green is very low expression, average expression is dark and higher expressions are red.

Transplant glomerulopathy is a pattern of chronic kidney allograft injury, which is linked to a poor transplant outcome. Recently it has been associated with chronic antibody mediated rejection, however, also non–immunological causes have been discussed [10, 35]. Clustering analysis of transcriptional data from the tubulointerstitial compartment resulted in two distinct expression patterns of lymphotoxins and related genes in patients with transplant glomerulopathy. One cluster was characterized by strong upregulation of LTα, TNF, LIGHT, HVEM, BTLA, CXCL13, CCR7 and CCL21 (Cluster 1), whereas the second pattern was distinctive by its robust expression of the LTβR, LTβ, TNF receptors 1 and 2, MADCAM and TROY (Cluster 2) (Fig 2). The clusters persisted when we used mRNA from the (laser dissected) glomerular compartment.

### mRNAs of the lymphotoxin family are upregulated/differentially expressed in human renal allografts with AR and IFTA

**Fig 3:**
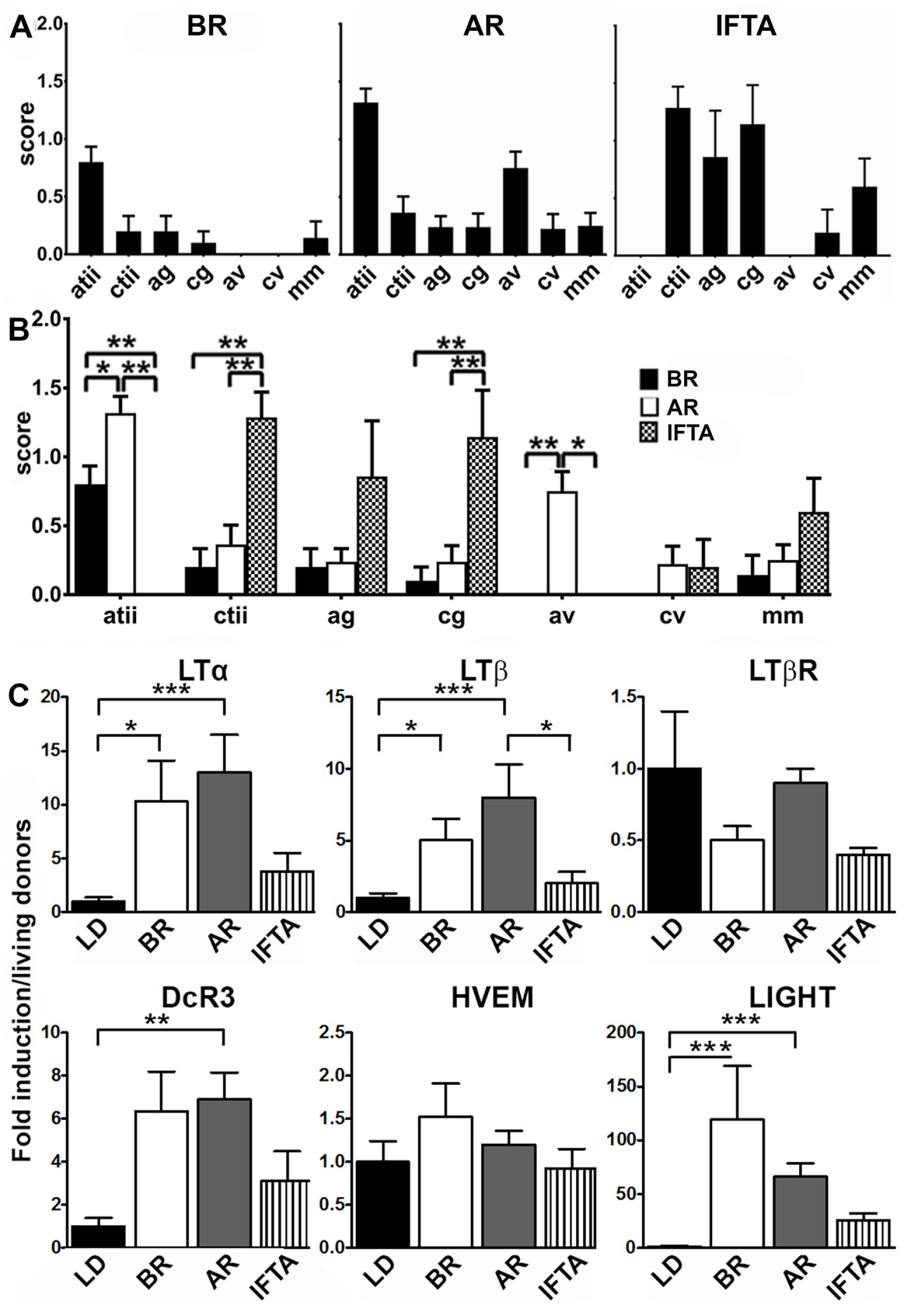
Upregulation of LT mRNAs in human renal allograft biopsies. (A) Detailed pathological changes of ERCB renal biopsy specimens in the different groups: BR (n = 10), AR (n = 22) and chronic injury (IFTA) (n = 7); atii acute tubulointerstitial inflammation, ctii chronic tubulointerstitial inflammation/interstitial fibrosis tubular atrophy, ag acute glomerulitis, cg chronic glomerulitis/transplant glomerulopathy, av acute intimal arteritis, cv chronic intimal arteritis/ arterial fibrous intimal thickening, mm mesangial matrix increase. BR borderline rejection, AR acute rejection, IFTA interstitial fibrosis/tubular atrophy. Displayed are mean score + SEM. (B) Displays significant differences between groups. (C) LTα, LTβ, LTβ receptor, DcR3 and LIGHT mRNA expression was quantified by quantitative RT-PCR (). Control biopsies were taken from living donors (LD) before implantation (n = 10). Significant upregulation is shown for Ltα, LTβ, DcR3 and LIGHT in borderline and AR. No differential regulation was observed for HVEM and the LTβR. There was a tendency towards higher expression of Ltα, LTβ, DcR3 and LIGHT mRNA in IFTA compared to controls, however results did not reach significance. The graphs show expression ratios of each gene normalized to 18 srRNA (* p< 0.05; **p < 0.01, *** p<0.001.

To confirm the transcription of components of the LT family in human renal allografts, we quantified mRNA expression by real-time RT-PCR in another series of 49 allograft biopsies. These included biopsies with borderline lesions (BR) (n = 10), AR (n = 22), and IFTA (n = 7). Control biopsies were taken from living donor derived grafts before implantation (n = 10). While biopsies from patients with borderline or AR where taken on average 3.8 and 5.3, biopsies from kidneys with IFTA were taken on average 73.5 months post-transplant (range 12–180 m) (Table 2). Detailed histopathological changes in each group are shown in Fig 3A. As expected, acute tubulointerstitial inflammation was significantly more pronounced in borderline and AR compared to the IFTA group. On the other hand chronic tubulointerstitial inflammation and chronic glomerulitis (transplant glomerulopathy) were significantly stronger in the IFTA group compared to AR and BR. Acute arteriitis was only present in AR, but not in borderline rejection or IFTA (Fig 3B).

As illustrated in Fig 3C, both, biopsies with borderline lesions and AR, demonstrated significant induction of LTα and LTβ mRNA. Interestingly, we observed no expression differences between borderline lesions and biopsies with AR. Biopsies from kidneys with IFTA demonstrated significantly less LTβ mRNA expression compared to biopsies with AR. The mRNA expression of LTβR did not demonstrate significant regulation, even though LTβR was prominently expressed (with Ct values between 23 and 29). However as observed in the microarray dataset, LIGHT mRNA was significantly upregulated in acute and borderline rejection, whereas its receptor HVEM was not. Also the TNF superfamily member DcR3 (TNFSFR6A), a soluble decoy receptor, which binds to LIGHT, TLA1 and Fas ligand (FasL) [36] displayed upregulation in borderline and AR, but not in chronic allograft injury. To assess whether cytokine (receptor) mRNA levels correlated with histopathological changes we performed Spearman’s correlation analysis. There was only a moderate correlation between active tubulitis/interstitial inflammation (atii) and the expression level of Ltβ mRNA (p<0.05) and active arteritis (av) and the expression of the LTβR mRNA (p<0.05) when patients from all groups were analyzed together (S1 Fig top left). In patients with borderline rejection there was no significant correlation between cytokine mRNA and histopathological changes (S1 Fig top right). In patients with acute rejection (AR) there was a significant negative correlation between atii and the expression of LTβR mRNA and a positive correlation between the expression of DCR3 mRNA and active glomerulitis (S1 Fig bottom left). Surprisingly, in patients with IFTA we observed a strong correlation between ctii and LTβ mRNA expression (S1 Fig bottom right).

### LTβ is expressed by tubular epithelial cells and infiltrating inflammatory cells in human renal allograft biopsies with AR and IFTA

**Fig 4:**
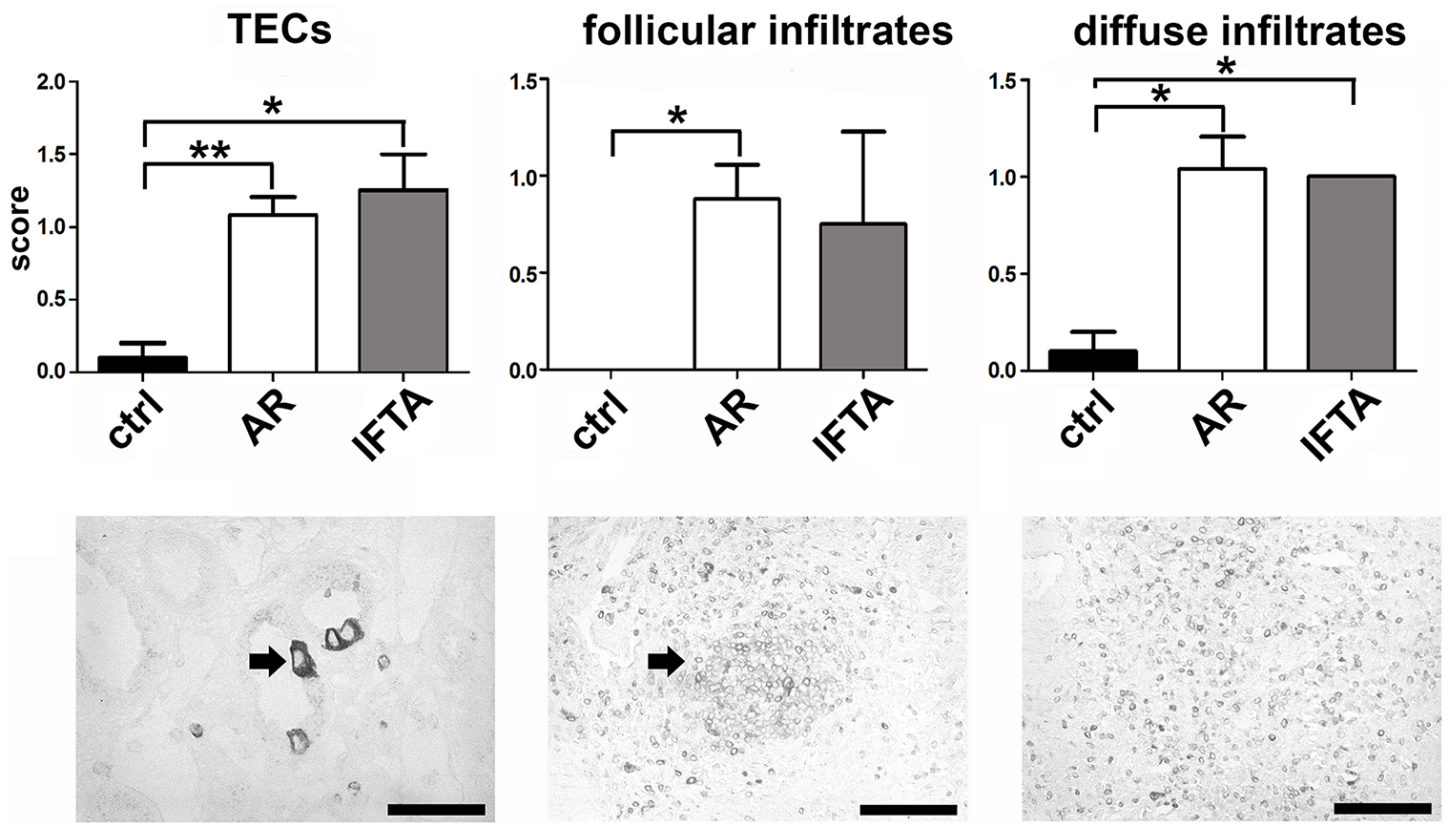
Semiquantitative expression of LTβ in human allograft biopsies. LTβ immunohistochemistry was performed in biopsies with acute rejection (AR), IFTA (IFTA) and controls (ctrl). Upper panel shows semiquantitative analysis of LTβ positivity in tubular epithelial cells (TECs; left), LTβ positivity in infiltrating inflammatory cells in follicular infiltrates (middle) and LTβ positivity in diffusely infiltrating inflammatory cells (right). The lower panel shows examples of LTβ positivity in TECs (left; arrow depicts positively stained tubular epithelial cell), follicular infiltrates (middle; arrow depicts follicular infiltrate) and diffusely infiltrating cells (right) (scale bar = 50 μM; * p < 0.05; ** p < 0.01, *** p < 0.001).

To verify expression of LTβ on the cellular level we performed immunohistochemistry using a monoclonal antibody against human LTβ on paraffin sections from kidney biopsies of 39 kidney transplant patients including ten pre-implant biopsies as controls (S4 Table). LTβ positivity was mostly confined to mononuclear inflammatory cells. These infiltrates ranged from diffuse accumulations in the tubulointerstitium to inflammatory cell aggregates with follicular appearance. Morphologically, the majority of positively stained cells resembled lymphocytes. In addition to infiltrating cells, we often observed strong immunoreactivity in the cytoplasm of tubular epithelial cells. Sometimes only one isolated cell within a tubular cross-section was positive, but at times also the entire tubular cross-section. (Fig 4). In the semiquantitative analysis, LTβ positivity in tubular epithelial cells was significantly more frequent in AR and IFTA compared to controls. Follicular infiltrates with Ltβ positivity were more frequent in AR compared to controls, whereas diffuse infiltrates with LTβ positivity were significantly more frequent in both AR and IFTA when compared to control biopsies. We did not observe a clear difference between biopsies with AR and biopsies with IFTA. In the implant biopsies, there was barely any immunoreactivity for LTβ (Fig 4).

### mRNAs of the lymphotoxin family are strongly upregulated in rejected mouse renal allografts

**Fig 5:**
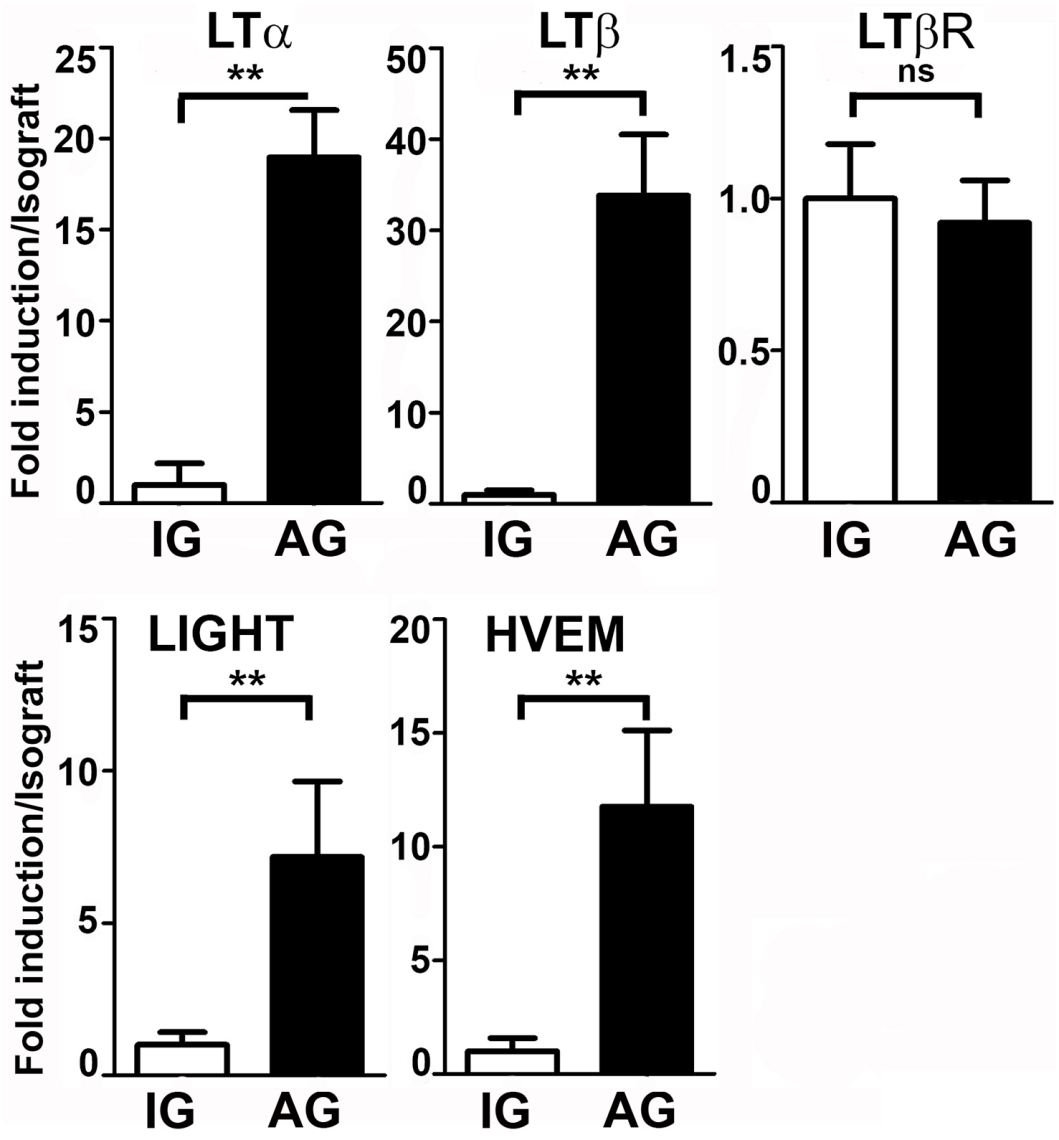
Quantification of lymphotoxin mRNAs in mouse renal allografts. Mean fold-induction of LTα, LTβ, LTβ receptor (all upper panel), HVEM and LIGHT mRNAs (lower panel) quantified by quantitative RT-PCR, demonstrated for full mismatched allografts (AG) compared to isografts (IG) (control) seven days post transplantation. A very significant induction was found for LTα, LTβ, HVEM, LIGHT, but no regulation was found for the LTβ receptor. (* p< 0.05, ** p < 0.01, *** p<0.001, ns = non significant.)

Finally, we investigated the expression of lymphotoxins in renal allograft rejection in a murine model. We transplanted kidneys from C57BL/6 mice into CBA (fully MHC mismatched allografts) or into C57BL/6 (isografts). Endogenous kidneys were used as controls. Kidneys were removed after seven days without any immunosuppressive treatment. Fig 5 illustrates results of the mRNA expression analysis of the lymphotoxin system. A highly significant induction was found for LTα, LTβ, HVEM and LIGHT, but again no change in expression was detected for the LTβ receptor mRNA. These data are consistent with our expression analysis from human kidneys, where also no regulation was found for the LTβ receptor.

## Discussion

Since LTs have an important function in various inflammatory processes we investigated the expression of members of the LT family and LT target genes in acute and chronic allograft injury. Analysis of transcriptomes from kidney grafts demonstrated robust upregulation of LTβ, LIGHT and one of its receptors, HVEM, as well as TNF receptors 1 and 2 mRNA in AR (Fig 1A). Interestingly, in chronic allograft injury/IFTA we observed a similar pattern (Fig 1B). The upregulation of transcripts controlled by the NF-κB signalling pathway, such as CXCL12 (SDF-1), CXCL13 (BLC), CCL19 (ELC) and BAFF in AR and IFTA (Fig 1C and 1D) strongly suggests activation of the LTα1β2-LTβR or LIGHT-LTβR axis since aside from CD40L-CD40 interaction and BAFF [37–40] LTβR activation is one of three major inducers of the alternate NF-κB pathway [41]. CD40L was neither upregulated in AR nor chronic allograft nephropathy and therefore is unlikely to be solely responsible for the assumed alternate NF-κB pathway triggering in this context. Because BAFF expression itself can be induced upon LTβR ligation via non-canonical NF-κB activation [20], it is difficult to dissect whether BAFF upregulation was a consequence or cause of the presumed activation of the alternate NF-κB pathway. LTα and TNFα mRNAs were not significantly increased in the transcriptome of grafts with AR and IFTA compared to nephrectomy controls which was unexpected, since lymphocytes as well as resident dendritic cells have been shown to express these cytokines [42–44]. LTβR mRNA did not show any differential regulation. This is in line with the previously described constitutive expression of the receptor in parenchymal cells and its promotor region suggesting a house keeping function [45]. Also earlier studies from our group in patients with various forms of glomerulonephritis, demonstrated that LTβR expression was unaltered in inflamed kidneys [15].

In a second experimental paradigm, mRNA expression patterns of members of the LT family and structurally related genes were assessed using cDNA arrays in patients with another chronic renal allograft injury, transplant glomerulopathy. Clustering analysis of mRNA expression from the tubulointerstitial compartment showed two distinct expression patterns. One cluster displayed strong upregulation of LTα, TNF, LIGHT, HVEM, BTLA, CXCL13, CCR7 and CCL21 (cluster 1), whereas the second pattern (cluster 2) was characterized by increased expression of the LTβR, LTβ, TNF receptors 1 and 2, MADCAM and TROY (Fig 2). The segregation of these two clusters could reflect different progression stages of transplant glomerulopathy or alternatively distinctive underlying pathologies characterized by engagement of a different subset of components of the LT and TNF system ultimately resulting in a similar histopathologic pattern. The clusters could also reflect different stages of chronic interstitial inflammation with formation of ectopic lymphoid follicles. Thaunat and others performed similar experiments in explanted renal allografts with terminal graft failure due to chronic rejection. They investigated the expression of several transcripts involved in ectopic lymphoid neogenesis such as LTα, β, LTβR, CXCL12, 13, CXCR4, 5, CCL19, 21 and CCR7. By hierarchical clustering, they identified three different clusters, which they attributed to different stages of lymphoid neogenesis in the allograft [46].

Other groups have shown expression of LTα in acutely rejecting kidney grafts [26, 47]. Using cDNA from the ERCB-KFB, we confirmed these findings and also detected robust upregulation of LTβ, and LIGHT in borderline rejection and AR when compared to controls from kidney biopsies of living donors before implantation (Fig 3). In allografts with IFTA we also noticed a trend towards higher expression of LTα, β and LIGHT, which, however, did not reach statistical significance (Fig 3). In line with the above findings, LTβR was again not differentially regulated. Interestingly, also the decoy receptor 3 (DcR3 /TNFRSF6B) message was significantly upregulated in AR. DcR3 is a secreted decoy receptor which binds to and blocks the biologic action of the ligands FasL and LIGHT [36, 48–50] but can also activate T cells via binding to TL1A [51]. It has recently been demonstrated to have negative prognostic value in CKD and HD patients [52]. Whether DcR3 is also a predictor of negative outcome in renal transplant patients needs to be determined. Interestingly, there was not a significant expression difference between borderline and AR with respect to the transcripts investigated. This could indicate that despite a morphologic difference on the light microscopic level acute and borderline rejection share features on the transcriptional level. Another explanation, which has been pointed out before, is that the Banff histological criteria are unreliable at the interface between borderline and acute rejection. Therefore, some of the cases diagnosed with borderline rejection might in fact have been cases of acute rejection [53,54].

In our study, most of the cytokine (receptor) mRNA levels in the different subgroups (borderline, AR and IFTA) did not significantly correlate with the degree of histopathological changes in the different renal compartments (S1 Fig). This does not indicate that the expression changes are insignificant. Several studies have shown that transcriptional upregulation of inflammatory genes or genes associated with injury do not correlate well with morphological changes in transplant biopsies, but often predict the outcome better than the histology [55, 56]. We cannot of course completely exclude that the lack of correlation is of methodological nature, since we extracted the histopathological changes from the biopsy reports and could not evaluate the changes directly from the histology slides.

To investigate the expression of LTβ on the protein level, we performed immunohistochemistry with an LTβ specific antibody. The analysis exhibited LTβ immunoreactivity in inflammatory cells and tubular epithelial cells in biopsies with AR and IFTA. In implant biopsies, we rarely observed LTβ immunoreactivity (Fig 4). In a previous study, we have also observed LTβ expression in TECs in biopsies from kidneys with different forms of glomerulonephritis [15]. The observed upregulation of LTβ in TECs suggests that tubular damage or TEC activation by cytokines from infiltrating cells leads to upregulation and release of LTβ by TECs, which may activate the LTβR on neighboring TECs and/or local immune cells such as DCs, modulating the inflammatory response. That TECs serve as regulatory cells in acute rejection—either amplifying or ameliorating inflammation—has been shown in previous studies [57]. Interestingly, we did not observe tubular expression of LTβ in implant biopsies. This indicates that even a severe ischemic stimulus (several hours of cold and minutes of warm ischemia) does not suffice to induce LTβ expression in TECs. As in kidneys with various forms of glomerulonephritis [15], expression of LTβ was observed in infiltrating mononuclear inflammatory cells diffusely within the tissue or in follicular or follicular-like infiltrates.

To investigate the expression of LTs in renal transplants in another species we employed an intrinsically very homogeneous model of acute renal allograft rejection using two fully mismatched inbred mouse strains [33]. In this model system, we were able to recapitulate our findings from human biopsy studies. Seven days after orthotopic renal grafting without immunosuppressive treatment histology of allografts showed severe acute tubulointerstitial rejection with interstitial edema, inflammatory infiltrates, tubulitis and signs of acute tubular injury. qPCR displayed significant induction of LTα, LTβ, HVEM and LIGHT mRNAs. Again LTβR was not differentially expressed (Fig 5). Whereas LTα has been detected in rat kidney transplants [25], to our knowledge, our study is the first showing upregulation of members of the LT family in rejected mouse kidney allografts. These findings indicate—at least with respect to the LT system—that mice react in a similar fashion than humans to engraftment with a foreign kidney. Given the ample disparities between the human and mouse immune system, this finding is critical regarding the translatability of results from studies in renal transplantation using this model system.

There are limitations to this study. We did not perform immunohistochemistry for LIGHT, LTα and HVEM, because in our experience, it is unreliable on archival paraffin embedded material. Therefore, apart from LTβ the observed changes were documented on the transcriptional level and we cannot make a statement in which renal compartment the expression changes occurred. It is likely, that at least part of the differential expression of the investigated transcripts was due to infiltrating cells. Why LTα mRNA was significantly upregulated in AR in the transcriptional analysis of the ERCB but not the GEO cohort is unclear. The fact that LTα was upregulated so strongly in AR *and* BR in the ERCB cohort, however, suggests that the findings are not coincidental. Finally, whether and which components of the LT system serve as positive or negative regulators in graft rejection cannot be deduced from our data and needs to be determined in future studies. Interfering with the LT system could be beneficial or counterproductive in solid organ transplantation. In a murine model of heart transplantation, blockade of LIGHT signaling lead to significantly prolonged graft survival [58]. Conversely, LTβR blockade in another model broke tolerance and caused inflammation and fibrosis in the cardiac allograft [59]. These equivocal results might relate to the fact that the network of interactions in the LT system is particularly complex as ligands can interact with different receptors and vice versa, making it difficult to anticipate the net effect of an intervention. In addition, manipulation of the LT system might not only result in blockade of specific processes such as inhibition of costimulation in T cell activation via LIGHT-HVEM but possibly also in global perturbance of immune effector functions as a consequence of secondary lymphoid organ disruption [13, 59].

To summarize, our results demonstrate that essentially all components of the LT system are expressed in human renal allografts with acute rejection and chronic injury as well as in acutely rejected mouse allografts. Furthermore, a subset of them are strongly upregulated in acute and chronic allograft injury. We therefore provide evidence for the involvement of the LT system in acute kidney rejection and chronic allograft injury. Yet, the exact functional and possibly diagnostic role of the LT system in these processes awaits further studies.

## Supporting information

S1 Table Clinical data of patients with acute allograft rejection (A), IFTA (B) and controls (C) included for analysis of lymphotoxin expression in renal graft transcriptomes. (DOCX)

S2 Table Clinical data of patients with transplant glomerulopathy (A) and controls (B) from which biopsy samples were utilized for microarray experiments. (DOCX)

S3 Table Clinical data of patients from the ERCB cohort with acute allograft rejection (A), borderline rejection (B), IFTA (C) and controls (live donors) (D) from which biopsy samples were used for RT-PCR experiments. (DOCX)

S4 Table Clinical data of patients with acute transplant rejection (AR), IFTA and controls from which graft biopsies were stained for LTβ. (DOCX)

S1 Fig Shows Spearman rank correlations of the cytokine (receptor) mRNA expression levels (normalized to 18s rRNA) with the histopathological changes described in Fig 3. There was a significant correlation between active tubulitis/interstitial inflammation (atii) and Ltβ (p < 0.05) and active arteritis (av) and the expression of the LTβR mRNA (p < 0.05) when patients from all groups were analyzed together (top left table). In patients with borderline rejection (top right table) there was no significant correlation observed between cytokine mRNA expression and histopathological changes. In patients with acute rejection (AR) (bottom left table) there was a significant negative correlation between atii and the expression of the LTβR mRNA and a positive correlation between the expression of the DCR3 mRNA and ag. In patients with IFTA (bottom right table) we observed a strong correlation between ctii and LTβ mRNA expression.
(TIF)
