# Supplementary material for: Lymphotoxin expression in human and murine renal allografts
Source: PLoS One. 2018 Jan 4;13(1):e0189396. doi: 10.1371/journal.pone.0189396 (PMC5754061; doi:10.1371/journal.pone.0189396)
Supplement: S1 Table — (DOCX) [file pone.0189396.s001.docx]

**A)**

| **Patient** | **Banff97** | **Graft type** | **Recipient age** | **Recipient sex** | **Creatinine μmol/l** |
| --- | --- | --- | --- | --- | --- |
| tx40 | IA | ND | 52 | m | nd |
| tx22 | IA | CAD | 41 | m | 187 |
| tx01 | IA | CAD | 47 | m | 230 |
| tx25 | IB | CAD | 54 | f | 466 |
| tx03 | IB | CAD | 22 | m | 161 |
| tx20 | IB | CAD | 35 | m | 171 |
| tx11 | IIA | CAD | 53 | m | 228 |
| tx50 | IIB | CAD | 48 | f | 328 |
| \| Mean (range) \|  \|  \| 1.27 \| 44.00 \| 6/2 \| 253.00 \| \| --- \| --- \| --- \| --- \| --- \| --- \| --- \| |  |  | \| 44 (22-54) \| 253.00 \| \| --- \| --- \| | 6/2 | 253 (161-466) |

**B)**

| **Patient** | **Banff97** | **IFTA** | **Graft type** | **Recipient age in y** | **Recipient sex** | **Creatinine in μmol/l** |
| --- | --- | --- | --- | --- | --- | --- |
| tx42 | CAN | I | CAD | 67 | m | 110 |
| tx02 | CAN | I | ND | ND | m | 120 |
| tx52 | CAN | I | ND | 27 | f | 121 |
| tx51 | CAN | I | ND | 55 | m | 267 |
| tx23 | CAN | II | CAD | 46 | f | 161 |
| tx31 | CAN | II | CAD | 47 | f | 167 |
| tx46 | CAN | II | CAD | 59 | m | 200 |
| tx44 | CAN | II | CAD | 49 | m | 182 |
| tx48 | CAN | II | CAD | 60 | m | 186 |
| tx13 | CAN | II | CAD | 60 | f | 332 |
| tx45 | CAN | II | CAD | 43 | m | 200 |
| tx55 | CAN | II | ND | 43 | f | 240 |
| tx49 | CAN | II | CAD | 45 | f | 298 |
| tx38 | CAN | III | CAD | 55 | m | 201 |
| tx24 | CAN | III | CAD | 45 | m | 210 |
| tx34 | CAN | III | CAD | 61 | m | 250 |
| tx29 | CAN | III | LD | 40 | m | 427 |
| tx07 | CAN | III | CAD | 39 | m | 495 |
| tx26 | CAN | III | ND | 33 | m | 572 |
| tx04 | CAN | III | CAD | 39 | m | ND |
| t32 | CAN | III | ND | 58 | m | 1000 |
| Mean (range) |  |  |  | 48.6 (24-67) | 15/6 | 287 (110-1000) |

**C)**

| **Patient** | **Age** | **Sex** | **Creatinine in μmol/l** |
| --- | --- | --- | --- |
| c01 | 61 | f | 65 |
| c03 | 71 | m | 61 |
| c04 | 28 | m | 75 |
| c05 | 37 | f | 36 |
| c06 | 44 | m | 88 |
| c10 | 64 | m | 91 |
| c11 | 82 | m | 113 |
| c13 | 76 | m | 88 |
| c14 | 74 | m | 107 |
| c15 | 60 | m | 93 |
| c16 | 17 | m | 114 |
| 281004c-7 | nd | nd | nd |
| Mean (range) | 55.8 (17–82) | 9/2/nd | 84.6 (36-114) |

m = male, f = female, nd = not determined, cad = cadaveric, CAN = chronic allograft nephropathy
