# Supplementary material for: Lymphotoxin expression in human and murine renal allografts
Source: PLoS One. 2018 Jan 4;13(1):e0189396. doi: 10.1371/journal.pone.0189396 (PMC5754061; doi:10.1371/journal.pone.0189396)
Supplement: S2 Table — (DOCX) [file pone.0189396.s002.docx]

**A)**

| **Patient** | **Recipient age in years** | **Recipient sex** | **Creatinine µmol/l** | **eGFR in ml/min** | **Proteinuria in g/d** |
| --- | --- | --- | --- | --- | --- |
| 1 | 68 | m | 336 | 14 | 4,6 |
| 2 | nd | nd | 193 | nd | 6,3 |
| 3 | nd | nd | 160 | nd | 4 |
| 4 | 41 | f | 143 | 37 | nd |
| 5 | 39 | m | 210 | 31 | nd |
| 6 | 39 | f | 269 | 17 | 0 |
| 7 | 49 | m | 168 | 38 | 1,8 |
| 8 | 51 | m | 228 | 26 | 0,1 |
| 9 | 37 | f | nd | nd | nd |
| 10 | 50 | f | 512 | 7 | 6 |
| 11 | 64 | m | 353 | 14 | 2,2 |
| 12 | nd | nd | 185 | nd | 7,6 |
| 13 | nd | nd | 302 | nd | 1 |
| 14 | 42 | m | 266 | 23 | 3 |
| **Mean** | **48** | **m/f/nd : 6/4/4** | **256** | **23** | **3,3** |
|  |  |  |  |  |  |

**B)**

| **Patient** | **Recipient age in years** | **Recipient sex** | **Creatinine µmol/l** | **eGFR in ml/min** | **Proteinuria in g/d** |
| --- | --- | --- | --- | --- | --- |
| 1 | 42 | f | nd | nd | nd |
| 2 | 61 | f | nd | nd | nd |
| 3 | 58 | f | nd | nd | nd |
| 4 | 25 | f | nd | nd | nd |
| **Mean** | **48** | **m/f: 0/4** | nd | nd | nd |

nd = not determined; m = male; f= female
