# Supplementary material for: Lymphotoxin expression in human and murine renal allografts
Source: PLoS One. 2018 Jan 4;13(1):e0189396. doi: 10.1371/journal.pone.0189396 (PMC5754061; doi:10.1371/journal.pone.0189396)
Supplement: S3 Table — (DOCX) [file pone.0189396.s003.docx]

| **Patient** | **Banff97** | **Graft type** | **Graft age in m** | **Recipient age in y** | **Recipient sex** | **Creatinine µmol/l** |
| --- | --- | --- | --- | --- | --- | --- |
| 1 | IB-IIA | cad | 2m | 53 | f | 217 |
| 2 | IIA | cad | 0m | 38 | m | 332 |
| 3 | IIA | cad | 2m | 47 | m | 253 |
| 4 | IIA | nd | nd | 38 | m | 248 |
| 5 | IIB | cad | 0m | 49 | m | 212 |
| 6 | IA | cad | 0m | 64 | m | 787 |
| 7 | IB | cad | 0m | 67 | m | 433 |
| 8 | IA | cad | 11m | 41 | m | 539 |
| 9 | IIA | LD | 0m | 44 | m | 813 |
| 10 | IB | cad | 2m | 64 | f | 707 |
| 11 | IIB | cad | 6m | 47 | m | 424 |
| 12 | IIA | cad | 4y (48m) | 71 | m | 455 |
| 13 | IIA | cad | 2m | 47 | f | 240 |
| 14 | IIA | cad | 0m | 49 | m | 720 |
| 15 | IB | cad | 0m | 61 | m | 456 |
| 16 | IA | cad | 0m | 54 | f | 909 |
| 17 | IA | cad | 2y (24m) | 49 | f | 177 |
| 18 | IB | cad | 8m | 47 | m | 440 |
| 19 | IIA | cad | 0m | 65 | m | 488 |
| 20 | IIB | cad | 0m | 61 | m | 637 |
| 21 | IIB | cad | 0m | 43 | f | 908 |
| 22 | IIA | nd | nd | 49 | m | 203 |
| Mean |  |  | 5.3 | 52.2 | m:f 16/6 | 217 |

**A)**

**B)**

| **Patient** | **Banff97** | **Graft type** | **Graft age in months** | **Recipient age in years** | **Recipient sex** | **Creatinine µmol/l** |
| --- | --- | --- | --- | --- | --- | --- |
| 1 | Borderline | LD | 24 | 40 | f | 159 |
| 2 | Borderline | LD | 4 | 25 | m | 136 |
| 3 | Borderline | cad | 0 | 50 | m | 725 |
| 4 | Borderline | cad | 3 | 60 | f | 262 |
| 5 | Borderline | cad | 1 | 57 | m | 228 |
| 6 | Borderline | cad | 0 | 38 | m | 194 |
| 7 | Borderline | cad | 4 | 47 | m | 360 |
| 8 | Borderline | cad | 0 | 38 | m | 195 |
| 9 | Borderline | cad | 1 | 66 | f | 150 |
| 10 | Borderline | cad | 1 | 64 | m | 919 |
| Mean |  |  | 3.8 | 40 | m/f : 7/3 | 332.8 |

**C)**

| **Patient** | **Banff97** | **IFTA** | **Graft type** | **Graft age in months** | **Recipient age in years** | **Recipient sex** | **Creatinine µmol/l** |
| --- | --- | --- | --- | --- | --- | --- | --- |
| 1 | CAN | II-III | cad | 180 | 42 | m | 280 |
| 2 | CAN | II | nd | 72 | nd | nd | 203 |
| 3 | CAN | II | cad | 120 | nd | nd | 239 |
| 4 | CAN | I-II | cad | 12 | 66 | f | 120 |
| 5 | CAN | II | cad | 108 | 43 | m | 410 |
| 6 | CAN | II | nd | 60 | 52 | nd | 548 |
| 7 | CAN | I | cad | 12 | 67 | m | 294 |
| 8 | CAN | I-II | nd | 24 | 71 | m | 258 |
| 9 | CAN | II-III | cad | 180 | 42 | m | 280 |
| 10 | CAN | II | nd | 72 | nd | nd | 203 |
| Mean |  |  |  | 73.5 | 52.0 | m/f : 5/1/4 na | 294 |

**D)**

| **Donor** | **Donor age in years** | **Donor sex** | **Creatinine µmol/l** |
| --- | --- | --- | --- |
| 1 | 35 | f | <97 |
| 2 | 56 | f | <97 |
| 3 | 41 | m | <97 |
| 4 | 62 | m | <97 |
| 5 | 27 | m | <97 |
| 6 | na | na | <97 |
| 7 | 56 | f | <97 |
| 8 | 70 | f | <97 |
| 9 | 53 | m | <97 |
| 10 | 28 | m | <97 |
| Mean | 49.1 | m/f : 5/4/1na | < 97 |

M = male, f = female, nd = not determined, cad = cadaveric, CAN = chronic allograft nephropathy, LD = living donation
