# Supplementary material for: Lymphotoxin expression in human and murine renal allografts
Source: PLoS One. 2018 Jan 4;13(1):e0189396. doi: 10.1371/journal.pone.0189396 (PMC5754061; doi:10.1371/journal.pone.0189396)
Supplement: S4 Table — (DOCX) [file pone.0189396.s004.docx]

| **Group** | **Patient** | **Recipient age in y** | **Recipient sex** | **Biopsy post transplantation in months** |
| --- | --- | --- | --- | --- |
| Control | 1 | 20 | m | 0,0 |
| Control | 2 | 39 | m | 0,0 |
| Control | 3 | 36 | f | 0,0 |
| Control | 4 | 54 | f | 0,0 |
| Control | 5 | 54 | m | 0,0 |
| Control | 6 | 65 | m | 0,0 |
| Control | 7 | 21 | m | 0,0 |
| Control | 8 | 57 | m | 0,0 |
| Control | 9 | 35 | m | 0,0 |
| Control | 10 | 4 | f | 0,0 |
|  | **Mean** | **38** | **m/f : 7/3** | **0,0** |
|  |  |  |  |  |
| IFTA | 1 | 43 | f | 8,1 |
| IFTA | 2 | 20 | m | 126,3 |
| IFTA | 3 | 77 | f | 6,2 |
| IFTA | 4 | 39 | m | 47,1 |
|  | **Mean** | **45** | **m/f : 2/2** | **46,9** |
|  |  |  |  |  |
| AR | 1 | 25 | m | 74,2 |
| AR | 2 | 43 | f | 13,2 |
| AR | 3 | 58 | f | 2,8 |
| AR | 4 | 51 | f | 10,8 |
| AR | 5 | 63 | m | 2,8 |
| AR | 6 | 49 | m | 68,4 |
| AR | 7 | 53 | ND | 11,0 |
| AR | 8 | 20 | f | 83,2 |
| AR | 9 | 28 | f | 0,2 |
| AR | 10 | 63 | m | 5,5 |
| AR | 11 | 58 | m | 0,3 |
| AR | 12 | 50 | m | 54,8 |
| AR | 13 | 48 | m | 7,3 |
| AR | 14 | 54 | f | 0,2 |
| AR | 15 | 60 | m | 41,3 |
| AR | 16 | 56 | f | 3,0 |
| AR | 17 | 54 | f | 1,5 |
| AR | 18 | 28 | f | 8,2 |
| AR | 19 | 65 | m | 1,6 |
| AR | 20 | 58 | m | 0,2 |
| AR | 21 | 52 | m | 9,6 |
| AR | 22 | 69 | m | 3,8 |
| AR | 23 | 37 | f | 0,4 |
| AR | 24 | 59 | m | 0,3 |
| AR | 25 | 29 | m | 1,3 |
|  | **Mean** | **49** | **m/f/nd : 14/10/1** | **16,0** |

M = male, f = female, nd = not determined, cad = cadaveric
