# Supplementary figures and images for: Lymphotoxin expression in human and murine renal allografts
Source: PLoS One. 2018 Jan 4;13(1):e0189396. doi: 10.1371/journal.pone.0189396 (PMC5754061; doi:10.1371/journal.pone.0189396)

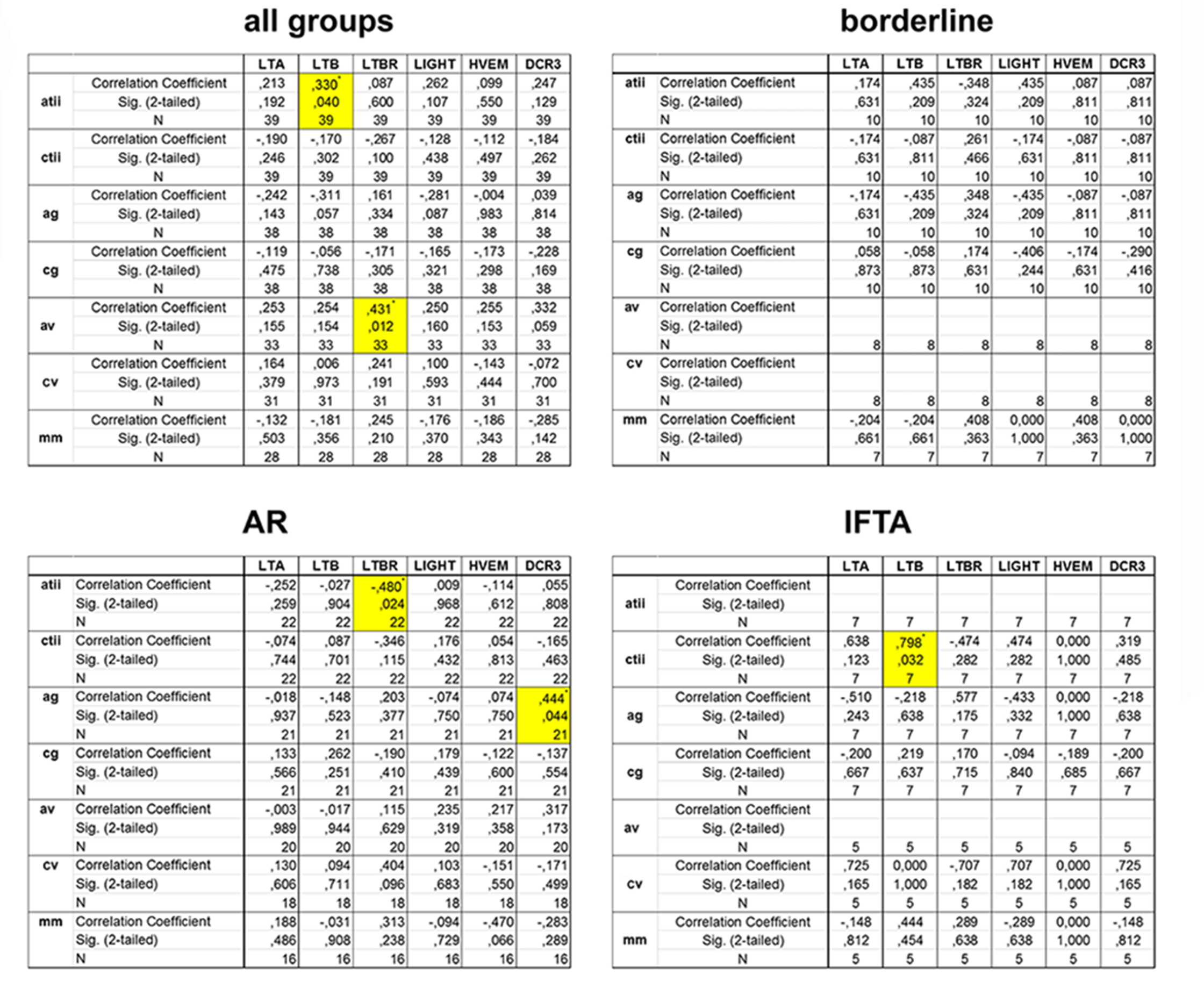

Supplement: S1 Fig — There was a significant correlation between active tubulitis/interstitial inflammation (atii) and Ltβ (p < 0.05) and active arteritis (av) and the expression of the LTβR mRNA (p < 0.05) when patients from all groups were analyzed together (top left table). In patients with borderline rejection (top right table) there was no significant correlation observed between cytokine mRNA expression and histopathological changes. In patients with acute rejection (AR) (bottom left table) there was a significant negative correlation between atii and the expression of the LTβR mRNA and a positive correlation between the expression of the DCR3 mRNA and ag. In patients with IFTA (bottom right table) we observed a strong correlation between ctii and LTβ mRNA expression. (TIF) [file pone.0189396.s005.tif]
